# Dual-band wide-angle metamaterial perfect absorber based on the combination of localized surface plasmon resonance and Helmholtz resonance

**DOI:** 10.1038/s41598-017-06087-1

**Published:** 2017-07-18

**Authors:** Changlei Zhang, Cheng Huang, Mingbo Pu, Jiakun Song, Zeyu Zhao, Xiaoyu Wu, Xiangang Luo

**Affiliations:** 1 State Key Laboratory of Optical Technologies on Nano-Fabrication and Micro-Engineering, Institute of Optics and Electronics, Chinese Academy of Sciences, P. O. Box 30, Chengdu, 610209 China; 2 University of Chinese Academy of Sciences, No. 19(A) Yuquan Road, Shijingshan District, Beijing, 100049 China

## Abstract

In this article, a dual-band wide-angle metamaterial perfect absorber is proposed to achieve absorption at the wavelength where laser radar operates. It is composed of gold ring array and a Helmholtz resonance cavity spaced by a Si dielectric layer. Numerical simulation results reveal that the designed absorber displays two absorption peaks at the target wavelength of 10.6 μm and 1.064 μm with the large frequency ratio and near-unity absorptivity under the normal incidence. The wide-angle absorbing property and the polarization-insensitive feature are also demonstrated. Localized surface plasmons resonance and Helmholtz resonance are introduced to analyze and interpret the absorbing mechanism. The designed perfect absorber can be developed for potential applications in infrared stealth field.

## Introduction

Recently, metamaterials (MMs) have attracted much attention owing to their unprecedented ability to manipulate electromagnetic (EM) wave. A great number of intriguing applications, such as electromagnetic cloaking^1, 2^, super lens^3, 4^, low-RCS materials^5–7^, polarization convertor^8, 9^, and perfect absorber^10–12^, have benefited from advances in metamaterial technology. Among these applications, the MMs absorbers are much significant in stealth field. To date, they have been realized and verified in most technologically relevant spectral range from microwave^10, 13–15^, mm-wave^16, 17^, THz^18, 19^, IR^20, 21^, to the visible^22–24^. Due to the inherent narrow bandwidth performance of the MMs absorbers, great efforts are made to accomplish multiband or broadband absorbers. There are several methods developed to extend the absorbing bandwidth, including the mixture of the different resonance structures^25, 26^, multi-layer structures^27^ and utilization of PIN diodes^28^. For the dual-band absorbing response, one of the most important parameters for the absorber is the frequency ratio (*f*_*u*_/*f*_*l*_), where *f*_*u*_ and *f*_*l*_ are the central frequencies of the higher and lower absorbing bands, respectively. Different frequency ratio would require different design approach. As a general method, a wide-band structure is employed to simultaneously cover the two bands, but it is only suitable for the absorber with a relatively small frequency ratio.

Laser radar is an important instrument in radar applications, playing a significant role in the area of target detection. There are two common wavelengths, 10.6 μm and 1.064 μm, where laser radar operates. To keep invisibility in front of the laser radar, the corresponding dual-band absorber is necessary, but the frequency ratio of the above two central points is too large (~10) for the wide-band absorber to simultaneously cover these two target points. In order to increase the frequency ratio of the dual-band absorber, different resonance structures in single- or multi-layer have been developed^18, 25, 29–32^, but the frequency ratio is limited to 3. It is still a challenge to achieve dual-band absorbers with a larger frequency ratio. Therefore, the objective of this paper is to achieve perfect absorption at the two operation wavelengths of the laser radar with such a large frequency ratio. A dual-band metamaterial absorber that utilizes a Helmholtz resonance cavity is proposed. Numerical simulation result shows that it can achieve perfect absorption at the two target wavelengths without other obvious absorption peaks between them. The polarization-insensitive feature is achieved based on the design of the symmetrical structure. In addition, the designed absorber is also demonstrated to keep high absorption efficiency as high as 90% at the large incident angle of 60°. The physical mechanism for dual-band absorption peak is discussed by examining the electric and magnetic field distributions. With the predominance of high absorptivity, polarization-insensitive feature and wide-angle absorbing property, this design can be developed for potential applications in infrared EM stealth field.

## Results and Discussions

**Figure 1:**
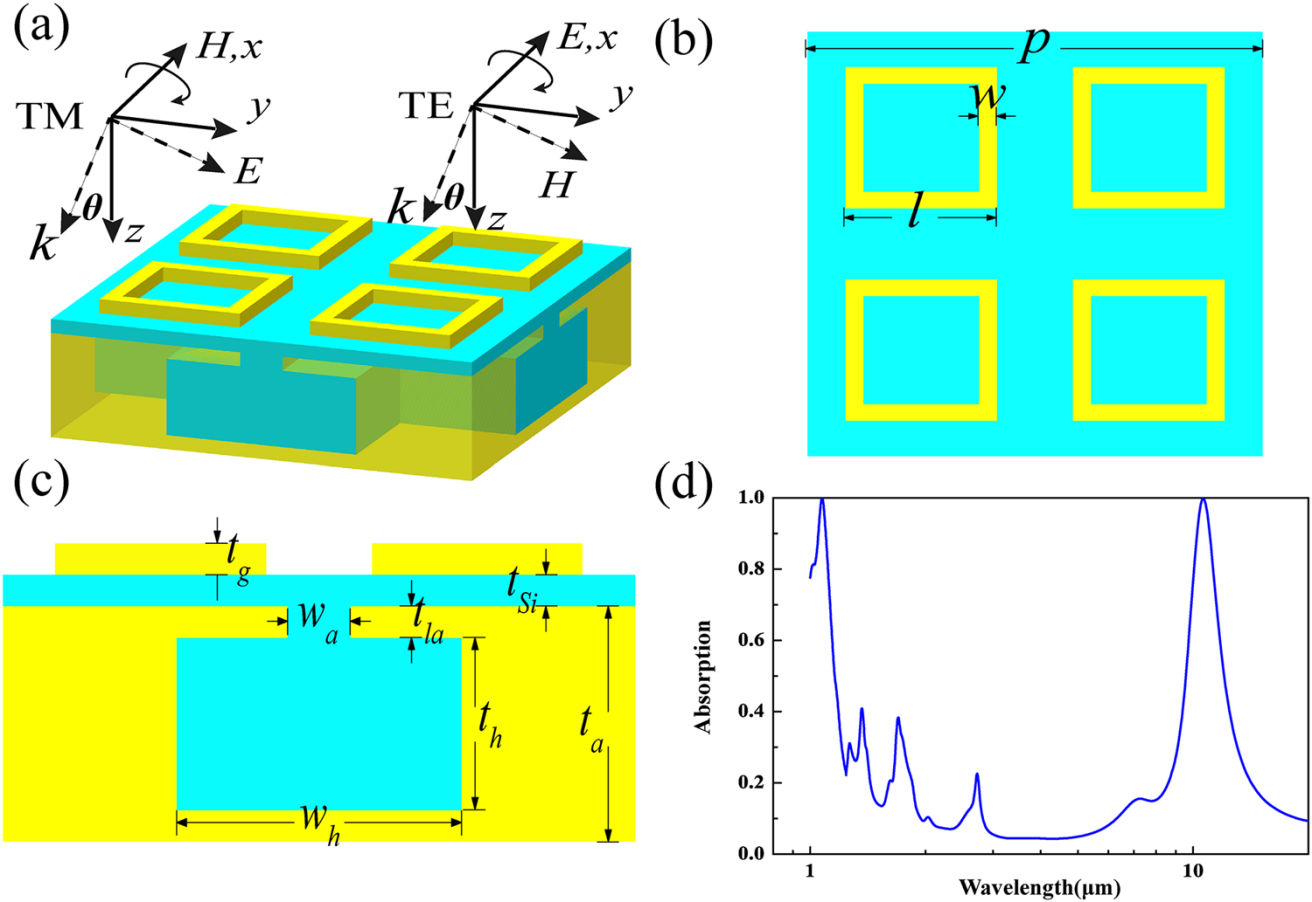
Geometrical model of the proposed dual-band perfect absorber. (**a**) 3D view. (**b**) Top view. (**c**) Side view. (**d**) Absorption spectrum of the designed absorber under normal incidence.

Figure 1a shows geometrical model of the proposed dual-band absorber. It is composed of a top metallic pattern layer and a resonance cavity spaced by a Si dielectric layer. The top metallic pattern layer is made of periodic gold ring arrays. As Fig. 1b shows, the period of the gold ring is *p*_*1*_ = p/2 = 0.5 μm, and its edge length and width are set to be *l* = 2*p*_*1*_/3 and *w* = 0.04 μm, respectively. The thickness of Si spacer is *t*_*Si*_ = 0.04 μm. The Helmholtz resonance structure filled by the Si dielectric is utilized to construct the resonance cavity because it is able to achieve perfect absorption^33^. The geometrical sizes of the Helmholtz cavity shown in Fig. 1c are optimized as follows: *p* = 1 μm, *t*_*a*_ = 0.3 μm, *t*_*la*_ = 0.04 μm, *t*_*h*_ = 0.22 μm, *t*_*g*_ = 0.017 μm, *w*_*a*_ = 0.09 μm, *w*_*h*_ = 0.45 μm.

**Figure 2:**
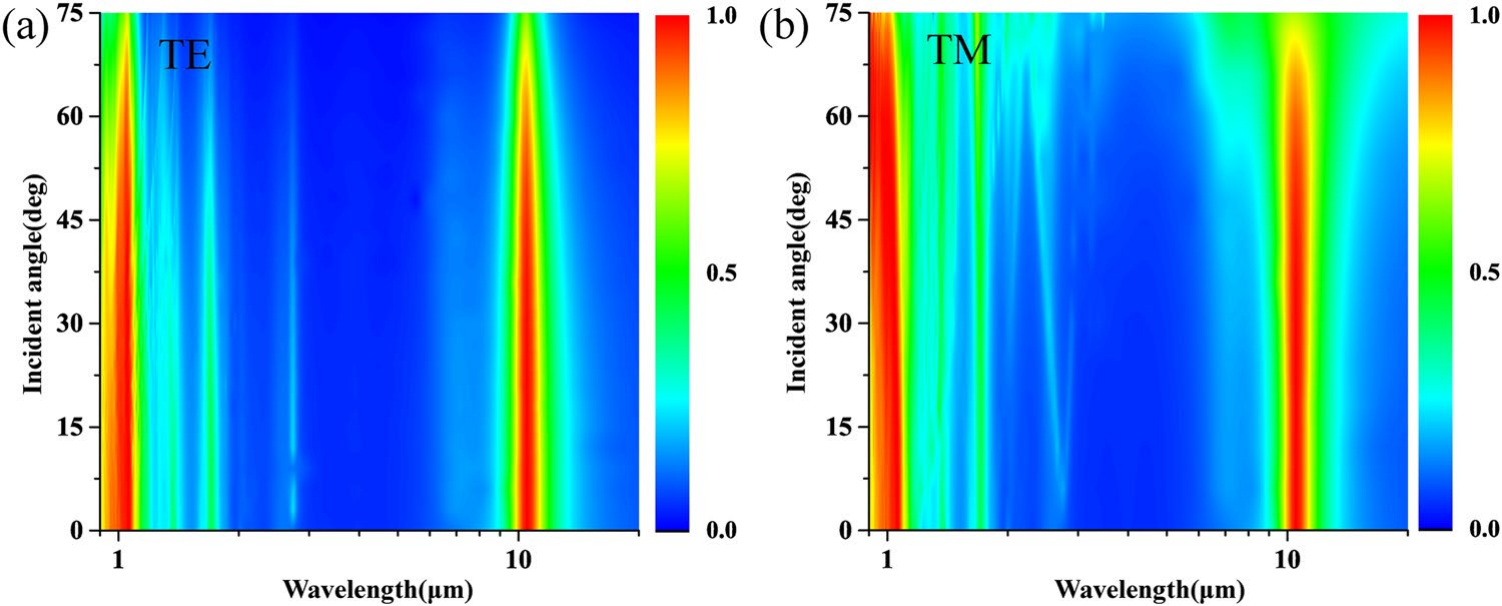
Absorption spectrum of the designed dual-band perfect absorber as a function of wavelength and the incident angle under different polarizations. (**a**) TE mode and (**b**) TM mode.

Numerical simulation is carried out to investigate absorption spectrum as depicted in Fig. 1d. It is seen that two perfect absorption peaks are realized at 1.064 μm and 10.6 μm where the absorptivity is as high as 99.916% and 99.754%, respectively. Between these two target frequencies, there are some other absorption peaks at 7.485 μm, 2.815 μm, 1.752 μm and 1.410 μm, but their corresponding absorption efficiencies are very weak, which are about 16.31%, 22.59%, 38.35% and 40.92%, respectively. The absorbing properties of the designed absorber under the oblique incidence are discussed as well. In the case of TE wave, there are two obvious absorbing bands around 1.064 μm and 10.6 μm, respectively, as seen in Fig. 2a. The proposed absorber can keep high absorption efficiency over a wide range of incident angle, and about 90% absorptivity is still remained even at a large oblique incident angle of 60° for both of the two absorption peaks. The absorbing property of this absorber in the TM case is shown in Fig. 2b, from which the similar wide-angle absorbing performance is still observed. Based on the above simulation results, the proposed absorber has been demonstrated to achieve perfect absorption at 10.6 μm and 1.064 μm without obvious absorption peaks between them. In addition, it has been verified to have polarization-insensitive feature and wide-angle absorbing capability.

**Figure 3:**
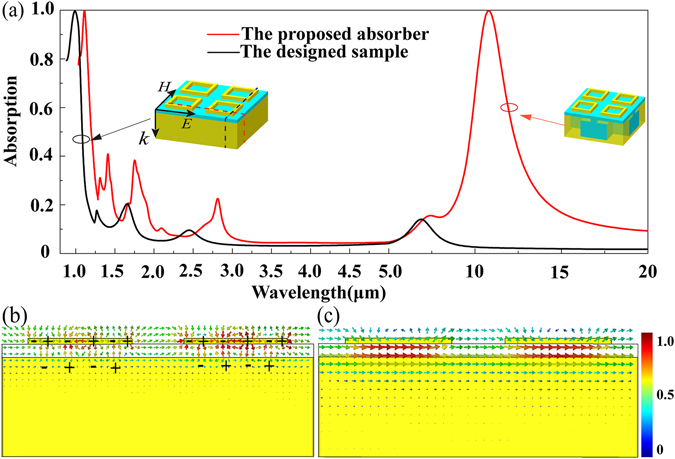
Sketch map interpreting the absorption peak at 1.064 μm. (**a**) Absorption spectrum of the designed absorber (red line) and a new sample (black line) under normal incidence. The red and black dotted lines on the new sample show the positions of cross section view for (**b**) Electric field distribution and (**c**) Magnetic field distribution at 0.989 μm, respectively.

To explain the physical mechanism of the absorption peak at 1.064 μm, another sample is designed, which consists of the identical gold ring arrays. Compared with the proposed absorber, only the original Helmholtz resonance cavity is replaced by the thick gold layer in the new sample, as seen in inset of Fig. 3a. From its absorption spectrum, it is seen that there is only one absorption peak at 0.989 μm with near-unit absorptivity. The red and black dotted lines on its geometrical model show the positions of cross section view for the electric field and magnetic field distributions, respectively. The electric field distribution is depicted in Fig. 3b. It shows obvious coupling between the gold rings and the thick gold layer, and the opposite electric charges are observed at corresponding position, indicating the production of localized surface plasmons resonance (LSPR)^27^. The magnetic field given in Fig. 3c is mainly distributed along the inner surface of the gold rings and the thick gold layer, which reveals that there is a strong magnetic resonance (MR) at the dielectric region along H-field direction^10^. We consider that the occurrence of MR could enhance the localized field at the resonance wavelength, and thus the near-unity absorption is realized by the enhanced LSPR^34^.

**Figure 4:**
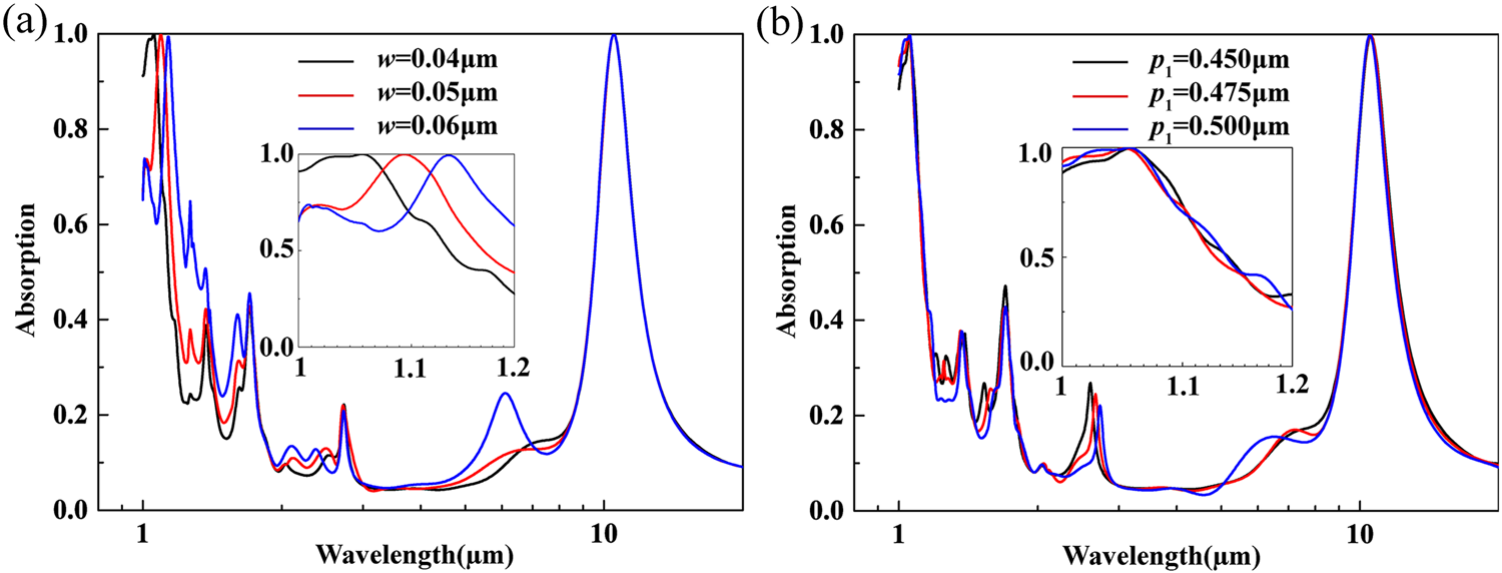
Response of absorption spectrum of the proposed absorber to different parameters of gold ring structure. (**a**) Period and (**b**) Width.

The physical mechanism described above can be further supported by the investigation of the gold rings’ width *w* and period *p*_*1*_ (*p*_*1*_ = *p/2*). The absorption spectra as a function of *w* is given in Fig. 4a. There is an obvious red shift with the increase of *w*, and the absorption peak occurs at the target position when *w* is 0.04 μm. The varying period has almost no influence in the absorption spectrum, as seen in Fig. 4b. The resonance wavelength for the absorption peak is invariable with the change of period *p*_*1*_, which is in accordance with the characteristics of LSPR and MR.

**Figure 5:**
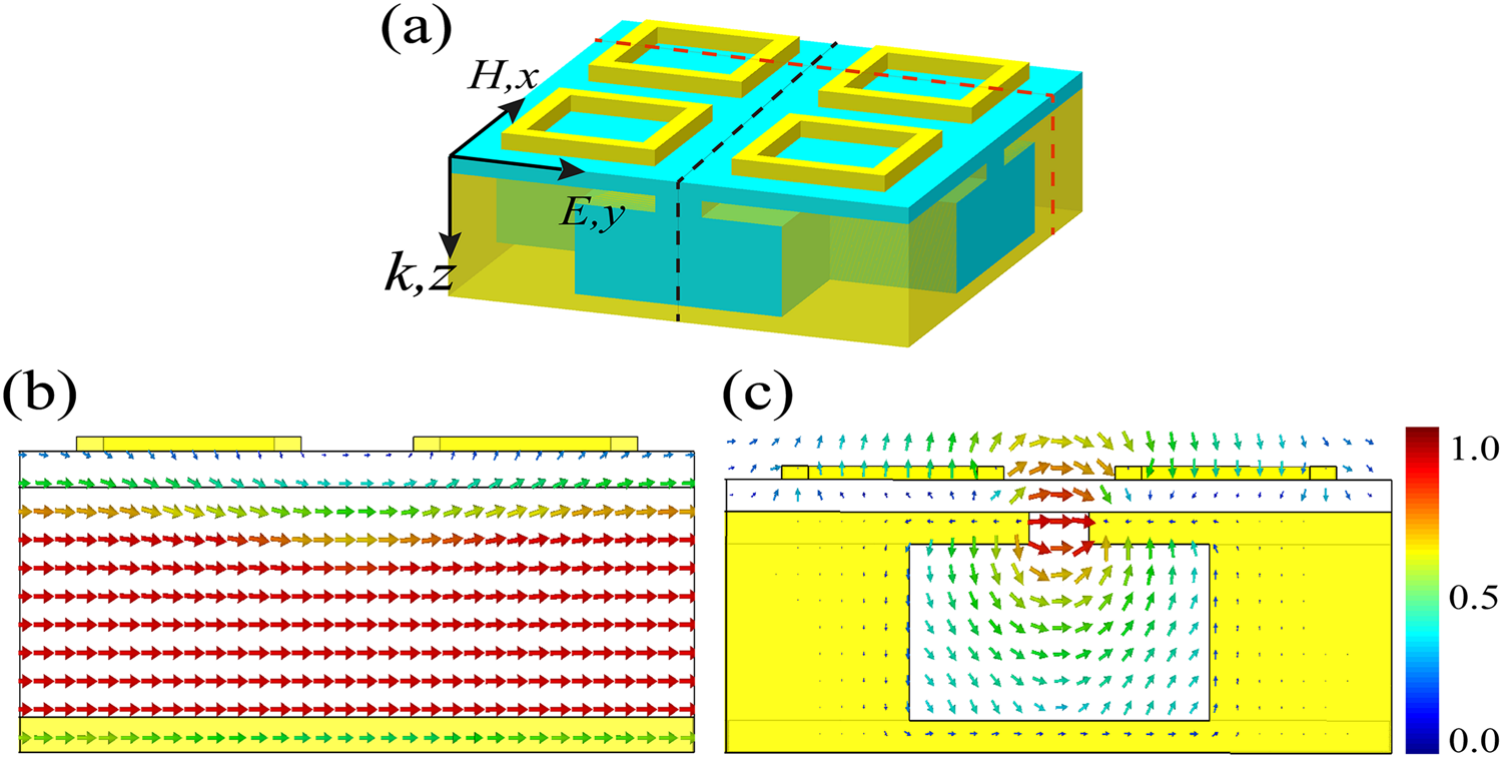
Magnetic field, electric field and effective capacitance model of the designed absorber at 10.6 μm. (**a**) The geometrical model of the absorber. The black dotted line shows the position of cross section view for (**b**) Magnetic field distribution. The red dotted line shows the position of cross section view for (**c**) Electric field distribution.

**Figure 6:**
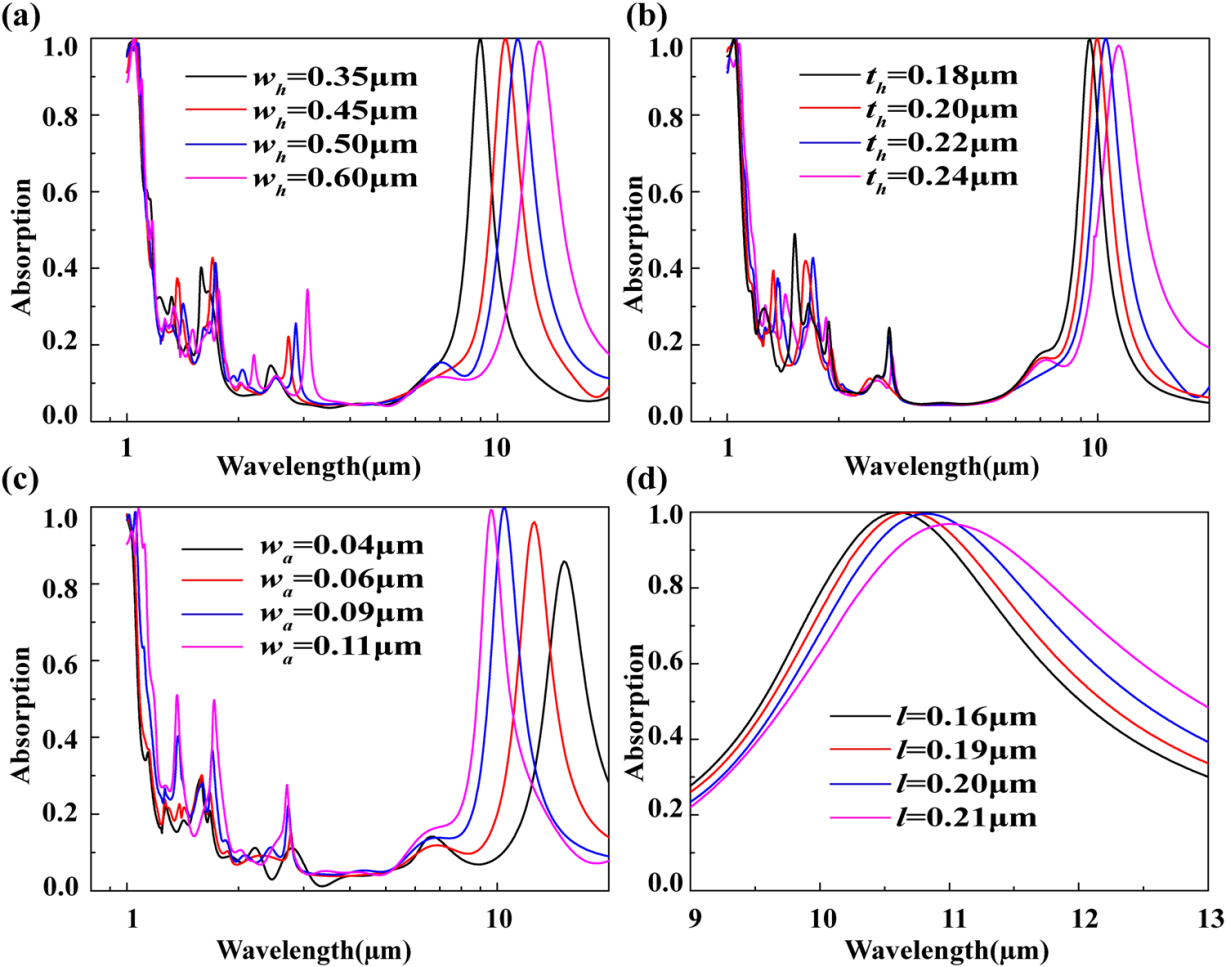
Absorption spectrum of the proposed absorber for different structure parameters. (**a**) Cavity width. (**b**) Cavity height. (**c**) Slit width. (**d**) Gold ring length.

It is still seen from Fig. 3a that the absorption peak at 10.6 μm disappeared when the cavity layer is replaced by the thick gold layer, which indicates that the absorption peak at 10.6 μm is closely associated with the Helmholtz resonance cavity. To further exploit the physical model of this observed absorption peak, the magnetic field and electric field distributions are investigated at 10.6 μm. In the process of calculation, the slits of the cavity are infinite along both *x*- and *y*-direction due to the periodic boundary, and then the Helmholtz Theorem can be suitable here^33^. The positions of cross section view for magnetic and electric field distribution are depicted in Fig. 5a, which are indicated by the black and red dotted lines, respectively. The magnetic field displayed in Fig. 5b is mainly localized in the Helmholtz resonance cavity along the incident H-field direction and its field intensity is almost consistent in the cavity, indicating that the cavity can be considered as an equivalent inductance that is proportional to the cavity width *w*_*h*_ and cavity height *t*_*h*_^33^. As Fig. 5c shows, most of the electric field energy is distributed around the slit of the cavity and some exists between the neighboring gold rings. These areas can be seen as a complex capacitance model that is associated with the slit width *w*_*a*_ and gold ring length *l* ^35^. Consequently, the *LC* resonance model can be adopted to investigate the Helmholtz resonance^33^, which can be further demonstrated by investigating the effect of the cavity width, cavity height, slit width and gold ring length on the absorption resonance wavelength. It is seen in Fig. 6a that there is an obvious shift of the absorption peak with the increase of the cavity width. When the value of *w*_*h*_ varies from 0.35 μm to 0.60 μm, the corresponding absorption resonance wavelength is shifted from 8.95 μm to 12.89 μm. The EM responses comply with the *LC* resonance model in which widening the cavity would increase the inductance, leading to the red shift of the absorption peak. The same phenomenon is also observed in Fig. 6b where increasing cavity height can realize the larger inductance. Figure 6c,d depict the frequency response of the absorption peak to different slit width and gold ring length, respectively. As Fig. 6c shows, the varying slit width has great influence in the resonance wavelength of the absorption peak that is reduced from 15.12 μm to 9.6 μm as the slit width is increased from 0.04 μm to 0.11 μm. Since the slit of the cavity can be equivalent as a capacitance model, the larger slit width gives rise to the smaller slit capacitance, resulting in the blue shift of the absorption peak. Similarly, the absorption peak position can be also fine-tuned through control of the gold ring length, as displayed in Fig. 6d. When the value of *l* is varied from 0.16 μm to 0.21 μm, the absorption peak has a weak frequency shift. That means the slit capacitance plays the great role in the whole complex capacitance model, while other capacitances caused by the introducing gold ring structure mainly achieve the fine-control of the absorption peak position. Such results are also in accordance with the electric field distribution given in Fig. 5c where the electric field intensity between the neighboring gold rings is obviously much weaker than that in the slit of the cavity. Compared with the previous Helmholtz resonance cavity^33^, a wider range of designs can be accessed in our absorber owing to the introducing gold ring structures that can influence the absorption peak. The above numerical analysis can well support the LC resonance model to explain the coupling between the incoming wave and the designed absorber in the Helmholtz resonance cavity.

## Conclusions

In conclusion, a dual-band wide-angle perfect absorber has been presented to keep invisibility in front of laser radar at the wavelength of 10.6 μm and 1.064 μm with the large frequency ratio. The corresponding absorptivity at the target frequencies can reach as high as 99.9%. It is still found that the proposed absorber is polarization-insensitive owing to its symmetrical structure and can achieve approximately 90% absorption efficiency even at the incident angle of 60 degree. The *LC* resonance theory is used to describe the near-unity absorption at 10.6 μm, while LSPR formed between the gold ring layer and the resonance cavity layer is proved to dominate the other absorption peak. It should be emphasized the design method is also suitable to be scaled to other frequency bands to achieve dual-band perfect absorption with the large frequency ratio.

## Methods

All the simulation results are obtained by using Frequency Domain Solver implemented in the commercial software CST STUDIO SUITE 2014. A Floquet port is utilized to produce a linear-polarized wave incident onto the cell and receives the reflection wave. Periodic boundary condition is applied for modeling infinite array. The absorption efficiency is calculated as *A*(*λ*) = 1−|S_11_|^2^ since there is no transmission for the use of a metallic ground plane. The unit cell of the proposed absorber is shown in Fig. 1a. The gold is described by Drude model in the infrared: $\varepsilon (f)={\varepsilon }_{\infty }-\frac{{f}_{p}^{2}}{f(f+i{f}_{c})},$ where *f* is the operation frequency, *ε*_∞_ is the offset value of permittivity, *f*_*p*_ is plasma frequency and *f*_*c*_ is collision frequency. In this case, the parameters in the Drude model for gold are listed as follows: *ε*_∞_ = 1, *f*_*p*_ = 1886.79 THz, and *f*_*c*_ = 14.528 THz^36^.
